# Lipid A structural diversity among members of the genus *Leptospira*

**DOI:** 10.3389/fmicb.2023.1181034

**Published:** 2023-05-25

**Authors:** Helena Pětrošová, Abanoub Mikhael, Sophie Culos, Alexandre Giraud-Gatineau, Alloysius M. Gomez, Matthew E. Sherman, Robert K. Ernst, Caroline E. Cameron, Mathieu Picardeau, David R. Goodlett

**Affiliations:** ^1^Department of Biochemistry and Microbiology, University of Victoria, Victoria, BC, Canada; ^2^University of Victoria Genome British Columbia Proteomics Center, University of Victoria, Victoria, BC, Canada; ^3^Institut Pasteur, Université Paris Cité, CNRS UMR 6047, Biology of Spirochetes Unit, Paris, France; ^4^Department of Microbial Pathogenesis, University of Maryland, Baltimore, MD, United States; ^5^Department of Medicine, Division of Allergy and Infectious Diseases, University of Washington, Seattle, WA, United States

**Keywords:** lipid A, *Leptospira*, mass spectrometry, structure-activity relationship, molecular typing, lipopolysaccharide (LPS), fast lipid analysis technique, pathogenicity

## Abstract

Lipid A is the hydrophobic component of bacterial lipopolysaccharide and an activator of the host immune system. Bacteria modify their lipid A structure to adapt to the surrounding environment and, in some cases, to evade recognition by host immune cells. In this study, lipid A structural diversity within the *Leptospira* genus was explored. The individual *Leptospira* species have dramatically different pathogenic potential that ranges from non-infectious to life-threatening disease (leptospirosis). Ten distinct lipid A profiles, denoted L1-L10, were discovered across 31 *Leptospira* reference species, laying a foundation for lipid A-based molecular typing. Tandem MS analysis revealed structural features of *Leptospira* membrane lipids that might alter recognition of its lipid A by the host innate immune receptors. Results of this study will aid development of strategies to improve diagnosis and surveillance of leptospirosis, as well as guide functional studies on *Leptospira* lipid A activity.

## 1. Introduction

Lipopolysaccharide (LPS) is one of the hallmark virulence factors of Gram-negative pathogens. It consists of three parts: O-antigen, core oligosaccharide antigen, and lipid A. The O-antigen is a polysaccharide exposed to the extracellular milieu, and its size and structural complexity delays the recognition of LPS by the host immune system and limits binding to host antibodies (Duerr et al., 2009; Domínguez-Medina et al., 2020). Core oligosaccharide consists of several different monosaccharide units, and connects O-antigen with lipid A. It contributes to stability of the outer membrane and it has antigenic properties (Silipo and Molinaro, 2010). Finally, lipid A, also known as endotoxin, anchors LPS to the outer leaflet of the outer membrane. Lipid A is the membrane anchor of LPS which attaches it to the outer leaflet of the outer membrane. It comprises two glucosamine sugars decorated with fatty acyl chains and terminal phosphate groups that can be further adorned with other functional moieties (Simpson and Trent, 2019).

The biological function of lipid A is dependent on its chemical structure. Bacteria modify their lipid A to adapt to changes in their surrounding environment (Needham and Trent, 2013; Simpson and Trent, 2019; Kawahara, 2021). These structural adaptations include, for example, modifications to the length and saturation of fatty acyl chains to overcome temperature shifts (Gunn and Ernst, 2007; Hassan et al., 2020) or addition of functional groups to gain resistance to antimicrobial peptides (Trent et al., 2001; Zhou et al., 2001), and are covered in great detail in recent reviews (Simpson and Trent, 2019; Kawahara, 2021). Lipid A is also a pathogen-associated molecular pattern. It interacts with the Toll-Like Receptor 4/Myeloid Differentiation protein 2 (TLR4/MD2) complex in a structure-dependent manner (Park et al., 2009; Ohto et al., 2012; Scott et al., 2017). The canonical hexa-acylated lipid A from *Escherichia coli* strongly activates TLR4/MD2, and is therefore highly endotoxic (Park et al., 2009). In contrast, tetra-acylated lipid A molecules are TLR4/MD2 antagonists (Baldridge and Crane, 1999; Deguchi et al., 2016). Similarly, lipid A molecules with two terminal phosphates are stronger TLR4/MD2 ligands than their monophosphorylated counterparts (Baldridge and Crane, 1999; Kong et al., 2012). Some pathogens, such as *Yersinia* and *Salmonella*, modify their lipid A structures accordingly to evade host inflammatory responses when establishing infection (Kawahara et al., 2002; Rebeil et al., 2004; Kong et al., 2011; Chandler et al., 2020).

*Leptospira* is a diverse group of bacteria comprising non-infectious free-living spirochetes, as well as pathogens that cause leptospirosis in a wide variety of hosts (Coburn et al., 2021). Unlike other spirochetes, all *Leptospira* possess LPS in their envelopes, and this molecule is central to the host immune responses to infection (Werts et al., 2001; Nahori et al., 2005; Viriyakosol et al., 2006; Murray et al., 2010; Srikram et al., 2011; Marcsisin et al., 2013). The lipid A structure has been established in serovars of the pathogenic *Leptospira* (Que-Gewirth et al., 2004; Eshghi et al., 2015; Novak et al., 2022). In contrast to the canonical di-glucosamine backbone of lipid A with amide- and ester-linked primary fatty acids (Simpson and Trent, 2019), the backbone of *Leptospira* lipid A comprises di-aminoglucose sugars, which results in linkage of all primary fatty acids through amide bonds (Que-Gewirth et al., 2004; Eshghi et al., 2015; Novak et al., 2022). In addition, the lipid A has only one terminal phosphate that is methylated; a structural feature that has not been described in any other bacterial species to date (Que-Gewirth et al., 2004; Simpson and Trent, 2019). These unique lipid A features are likely involved in the inability of *Leptospira* lipid A to bind to human TLR4/MD2 (Werts et al., 2001). Similar to other bacterial pathogens, *L. interrogans* modify their lipid A structure to adapt to temperature shifts (Gunn and Ernst, 2007; Eshghi et al., 2015).

Given the enormous diversity of the *Leptospira* genus (Vincent et al., 2019), the structural diversity of its lipid A is curiously understudied (Patra et al., 2015; Vanithamani et al., 2021; Novak et al., 2022). *Leptospira* are fastidious bacteria that grow slowly in rich and complex culturing media supplemented with host factors (Zuerner, 2005). The traditional protocols for lipid A extraction that require large volumes of bacterial culture are therefore likely the cause of this knowledge gap. To circumvent these limitations, we employed a rapid protocol for lipid A structural characterization, FLAT*^n^* (Leung et al., 2017; Sorensen et al., 2020; Yang et al., 2022a), that allowed us to utilize an estimated equivalent of 10^7^ *Leptospira* cells in 1 ml volume per assay. We examined lipid A mass spectral profiles, from which representative structures were proposed, in 31 *Leptospira* species from different phylogenetic groups. This work therefore represents the first comprehensive comparison of lipid A structure in virulent versus nonvirulent *Leptospira* species.

## 2. Materials and methods

### 2.1. *Leptospira* species

*Leptospira* species used in this study are listed in Table 1. *Leptospira* were grown in the Ellinghausen–McCullough–Johnson–Harris (EMJH) medium, as modified by Ellis and Thierman (EMJH T80/T40/LH); medium was prepared without the addition of rabbit serum and superoxide dismutase (Ellis and Thiermann, 1986; Zuerner, 2005). Cultures were kept at 30°C and shaking at 100 rpm. For all experiments, *Leptospira* species were grown in biological triplicates to mid-logarithmic phase (approximately 5× 10^8 cells/ml), as assessed by density and motility under a dark-field microscope (Zuerner, 2005).

**Table 1:** Reference *Leptospira* species used in this study.

Species	Strain	Group	Origin	Virulence (hamster)	Reference
*L. interrogans*	L495	P1*hv*	Human; Manila, Philippines	Yes	Koizumi and Watanabe (2003)
*L. mayottensis*	200901116	P1*hv*	Human; Mayotte	Yes	Bourhy et al. (2014)
*L. noguchii*	201102933	P1*hv*	Human; Guadeloupe	Yes	Vincent et al. (2019)
*L. santarosai*	LT821	P1*hv*	*Proechimys semispinosus* (spiny rat); Panama Canal Zone	Yes	Yasuda et al. (1987)
*L. weilii*	14535	P1*hv*	Human; Laos	Yes	Vincent et al. (2019)
*L. adleri*	M7A	P1*lv*	Water; Mayotte	ND	Vincent et al. (2019)
*L. ainazelensis*	201903074 10/E/19	P1*lv*	Water through (cow breeding); Aïn Azel, Algeria	ND	Korba et al. (2021)
*L. dzianensis*	M12A	P1*lv*	Water; Dziani, Mayotte	ND	Vincent et al. (2019)
*L. gomenensis*	KG8-B22	P1*lv*	Soil; Kaala-Gomen, New Caledonia	ND	Vincent et al. (2019)
*L. tipperaryensis*	GWTS1	P1*lv*	*Crocidura russula* (greater white-toothed shrew); Tipperary, Ireland	ND	Nally et al. (2016)
*L. fluminis*	SCS5	P2	Soil; Sungai Congkak, Malaysia	ND	Vincent et al. (2019)
*L. haakeii*	ATI7-C-A2	P2	River bank; Unia, New Caledonia	ND	Thibeaux et al. (2018)
*L. hartskeerlii*	MCA1-C-A1	P2	Soil; Ponerihouen, New Caledonia	ND	Thibeaux et al. (2018)
*L. langatensis*	SSW18	P2	Water; Sungai Congkak, Malaysia	ND	Vincent et al. (2019)
*L. licerasiae*	VAR010	P2	Human; Iquitos, Peru	No	Ricaldi et al. (2012)
*L. neocaledonica*	ES4-C-A1	P2	River bank; Koné, New Caledonia	No	Thibeaux et al. (2018)
*L. perolatii*	FH1-B-B1	P2	River bank; Touho, New Caledonia	No	Thibeaux et al. (2018)
*L. selangorensis*	SCW17	P2	Water; Sungai Congkak, Malaysia	ND	Vincent et al. (2019)
*L. venezuelensis*	CLM-U50	P2	*Rattus norvegicus* (rat); Venezuela	ND	Puche et al. (2018)
*L. bandrabouensis*	201601111 M10A	S1	Water; Bandraboua, Mayotte	ND	Vincent et al. (2019)
*L. biflexa*	Patoc 1	S1	Water; Italy, France	No	Picardeau et al. (2008)
*L. bourretii*	201800280 PZF7-6	S1	Soil; Nouméa, New Caledonia	ND	Vincent et al. (2019)
*L. bouyouniensis*	201601297 M1A	S1	Water; Bouyouni, Mayotte	ND	Vincent et al. (2019)
*L. harrisiae*	201602189 FH2-B A1	S1	River bank; Touho, New Caledonia	ND	Thibeaux et al. (2018)
*L. kanakyensis*	201800292 TK5-11	S1	Soil; Koné, New Caledonia	ND	Vincent et al. (2019)
*L. montravelensis*	201800279 PZF5-3	S1	Water; Nouméa, New Caledonia	ND	Vincent et al. (2019)
*L. mtsangambouensis*	201601298 M2A	S1	Water; Mtsangamboua, Mayotte	ND	Vincent et al. (2019)
*L. noumeaensis*	201800287 PZF14-4	S1	Water; Nouméa, New Caledonia	ND	Vincent et al. (2019)
*L. idonii*	201300427 DSM26084; Eri-1	S2	Water; Fukuoka, Japan	No	Saito et al. (2013)
*L. kobayashii*	E30	S2	Soil; Gifu, Japan	ND	Masuzawa et al. (2019b)
*L. ryugenii*	YH101	S2	Water; Shizuoka, Japan	ND	Masuzawa et al. (2019a)

Virulence column refers to the golden Syrian hamster model of leptospirosis. ND, not experimentally determined.

### 2.2. Fast lipid analysis technique (FLAT)

Lipid A structural analyses were performed using FLAT (Sorensen et al., 2020) and its tandem-MS version FLAT*^n^* (Yang et al., 2022a). Five milliliter of logarithmic *Leptospira* culture was centrifuged at 4,000x g for 15 min. Resulting pellets were washed twice with 1 mL of phosphate buffered saline (Sigma Aldrich, St. Luis, MO, USA), and resuspended in 200 μL of MS-grade water (Fisher Chemical, Hampton, NH, USA). One microliter of the sample was spotted on a MALDI plate (MFX μFocus plate 12×8 c 2,400 μm 0.7 T; Hudson Surface Technology, Inc., South Korea) and air dried. One microliter of the FLAT extraction buffer (0.2 M citric acid, 0.1 M sodium citrate in MS-grade water; both from Fisher Chemical) was added on the top of each sample. MALDI plate was placed in an in-house made humidifier chamber and incubated at 110°C for 30 min. Plate was gently washed with MS-grade water for approximately 30s and let air dry. Finally, 1 μL of norharmane matrix (Sigma Aldrich) was spotted on the top of each sample and let dry. Norharmane matrix was prepared at 10 mg/mL in 2:1 v/v MS-grade chloroform and methanol (both from Fisher Chemical).

### 2.3. MALDI MS analysis of lipid A

Mass spectra were obtained on a timsTOF *flex* MALDI-2 instrument (Bruker, Bremen, Germany) in the negative ion mode. Instrument was calibrated before each experiment in an electrospray mode by a direct infusion of the Agilent Calibration mix (Agilent Technologies, Santa Clara, CA, USA). Tandem MS analyses were performed with the following settings: 3,000 shots/spot on average, collision energy: 110–120 eV, isolation width: *m/z* 4, collision RF: 1,000 Vpp, transfer time: 110 μs and prepulse storage: 11 μs. To detect product ions in the low range *m/z*, the collision RF and transfer time were changed to 300 Vpp and 30 μs, respectively. Data were analyzed using mMass v5.5.0 (Strohalm et al., 2010) and Compass Data Analysis v 6.0 (Bruker). Fragmentation patterns of predicted lipid A structures were confirmed in ChemDraw v18.0 (PerkinElmer Informatics, Waltham, MA, USA). Theoretical isotopic distributions were predicted using Peak-by-Peak Metabolomics software v 2022.8.0 (Spectroswiss, Lausanne, Switzerland).

## 3. Results and discussion

Recent advances in the field of *Leptospira* genomics led to identification of 68 reference *Leptospira* species, and their reclassification into four distinct phylogenetic subclades (Vincent et al., 2019; Korba et al., 2021). The P1 subclade encompasses species formerly known as “pathogens,” the P2 subclade comprises species formerly known as “intermediates,” and finally, the S1 and S2 subclades encompass non-infectious saprophytic species. The P1 subclade is further divided into P1 high virulence (P1*hv*) and P1 low virulence (P1*lv*) groups. *Leptospira* species most frequently involved in human disease, such as *L. interrogans* and *L. noguchii*, belong to the P1*hv* group, whereas species with no/unknown pathogenic potential cluster to the P1*lv* group (Vincent et al., 2019). Although the differences between the individual subclades are clear on the genome level, additional knowledge on phenotypic differences is warranted to fully understand the pathogenesis of leptospirosis. Here we examined clade-specific differences in the structure of lipid A, the hallmark virulence factor of bacterial pathogens.

### 3.1. Ten unique lipid A profiles were detected between the individual *Leptospira* subclades

**Figure 1:**
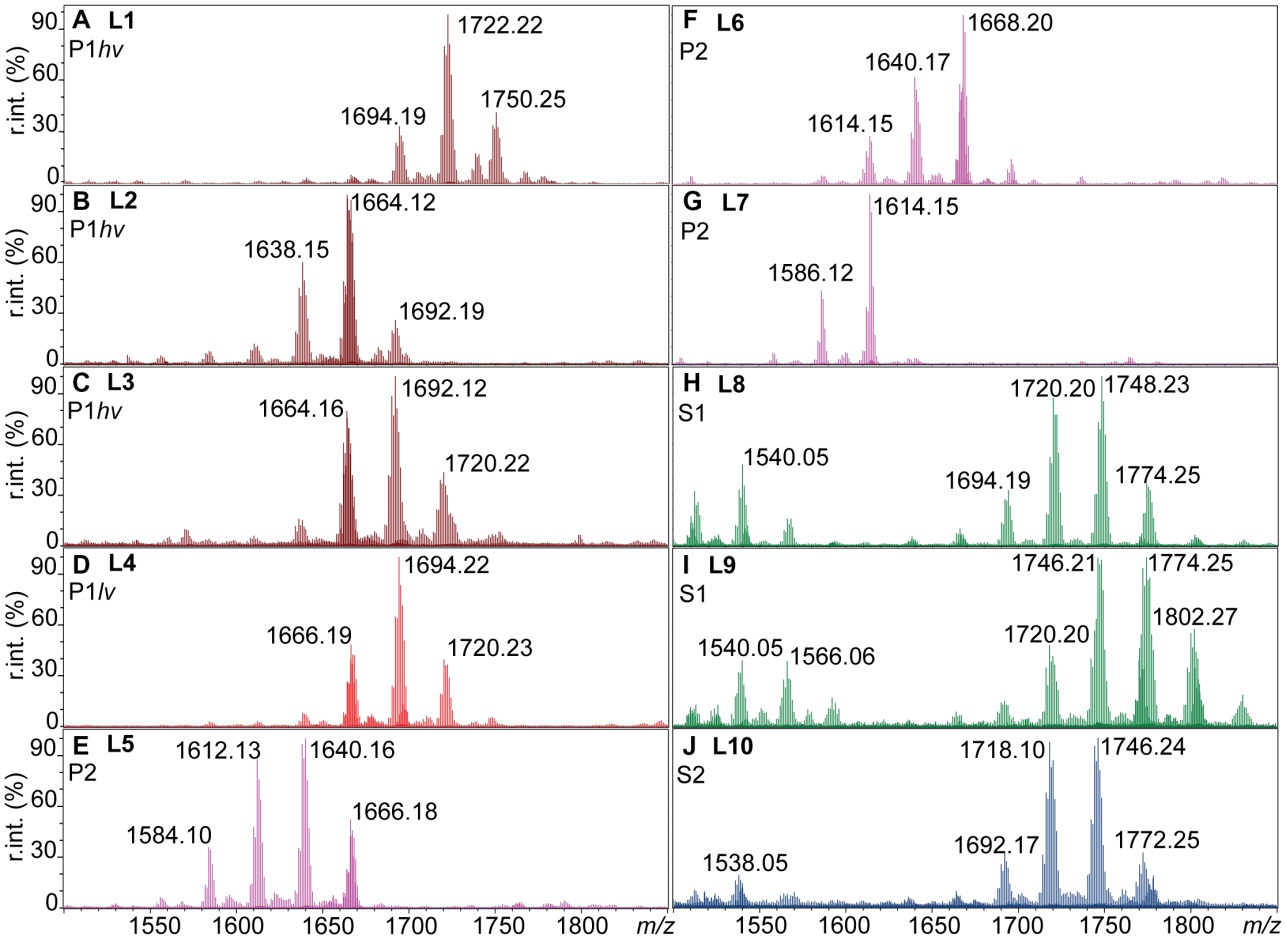
Ten lipid A profiles identified within the individual *Leptospira* subclades (L1-L10). One microliter of logarithmic cell suspensions in MS-grade water was spotted on a MALDI plate and subjected to FLAT (Sorensen et al., 2020). MS1 scans were acquired in the negative ion mode, relative intensities (r. int.) are shown. **(A–C)** P1*hv* group (dark red). **(D)** P1*lv* group (light red). **(E–G)** P2 subclade (purple). **(H,I)** S1 subclade (green). **(J)** S2 subclade (blue). Lipid A profiles of the individual *Leptospira* species can be found in Supplementary Figures S1–S3, and the information is summarized in Table 2.

The negative ion mass spectra of lipid A from 31 *Leptospira* species were examined by FLAT (Sorensen et al., 2020). This included five P1*hv*, five P1*lv*, nine P2, nine S1, and three S2 species (Table 1). In total, 10 different lipid A profiles denoted L1-L10 were detected across the examined species (Figure 1; Table 2). In the P1*hv* group, *L. interrogans*, *L. noguchii,* and *L. weilii* shared the L1 profile (Figure 1A), and L2 and L3 profiles were detected in *L. mayottensis* and *L. santarosai*, respectively (Figures 1B,C). The P1*lv* group was homogenous; all P1*lv* species shared the L4 profile (Figure 1D). In the P2 subclade, 7 out of the 9 species shared the L5 lipid A profile with *L. licerasiae* (Figure 1E). The L6 profile was detected in *L. fluminis* (Figure 1F) and the L7 profile in *L. perolatii* (Figure 1G). The lipid A profiles of S1 and S2 species were very similar to each other. Seven out of nine S1 species shared the L8 profile with the model saprophytic species *L. biflexa* (Figure 1H). *L. noumeaensis* and *L. kanakyensis* displayed the L9 phenotype (Figure 1I). Finally, the L10 profile was detected in all S2 species (Figure 1J). The individual lipid A profiles of all examined *Leptospira* species can be found in Supplementary material (Supplementary Figures S1–S3).

**Table 2:** Lipid A profiles identified in the individual subclades.

Subclade	Profile	Incidence	Lipid A ions (*m/z*)	*Leptospira* species
**P1*hv***	**L1**	(3/5)	1,694, **1,722** and 1,750	*L. interrogans*, *L. noguchii* and *L. weilii*
	**L2**	(1/5)	1,638, **1,664** and 1,692	*L. mayottensis*
	**L3**	(1/5)	1,664, **1,692** and 1,720	*L. santarosai*
**P1*lv***	**L4**	(5/5)	1,666, **1,694** and 1,720	*L. adleri*, *L. ainazelensis*, *L. dzianensis*, *L. gomenensis*, and *L. tipperaryensis*
**P2**	**L5**	(7/9)	1,584, 1,612, **1,640** and 1,666	*L. haakeii*, *L. hartskeerli*, *L. langatensis*, *L. licerasiae*, *L. neocaledonica, L. selangorensis*, and *L. venezuelensis*
	**L6**	(1/9)	1,614, 1,640 and **1,668**	*L. fluminis*
	**L7**	(1/9)	1,586 and 1,614	*L. perolatii*
**S1**	**L8**	(7/9)	1,540, 1,694, 1,720, **1,748** and 1,774	*L. bandrabouensis*, *L. biflexa*, *L. bouyouniensis*, *L. bourrettii*, *L. harrisiae*, *L. mtsangambouensis*, and *L. montravelensis*
	**L9**	(2/9)	1,540, 1,566, 1720, 1746, **1774** and 1802	*L. noumeaensis* and *L. kanakyensis*
**S2**	**L10**	(3/3)	1,538, 1,692, 1720, **1746** and 1772	*L. idonii*, *L. kobayashii*, and *L. ryugenii*

Numbers in brackets correspond to the number of interrogated *Leptospira* species with the corresponding lipid A profile. The most common lipid A profile is listed first for each subclade; base peak ions are highlighted in bold.

There was no obvious association between the origin of the examined *Leptospira* species and their lipid A profiles. For example, all but one examined P1*lv* species and all S1 species were isolated from water and soil environments (Table 1), yet their lipid A profiles were different (Figure 1). Presence of lipid A modifications that could aid survival of *Leptospira* in water and soil environments cannot be excluded. However, environment-induced lipid A modifications are often transient (Rebeil et al., 2004; Li et al., 2012) and unlikely to be carried over to bacteria grown under conditions where these modifications are not required. At the growth conditions used in this study (modified EMJH T80/T40/LH, 30°C, and shaking), the strongest association was observed between the lipid A profiles of the individual *Leptospira* species and their phylogenetic classification (Figure 1; Table 2).

### 3.2. *Leptospira* lipid A profiles were complex, displaying high intraspecies heterogeneity

**Figure 2:**
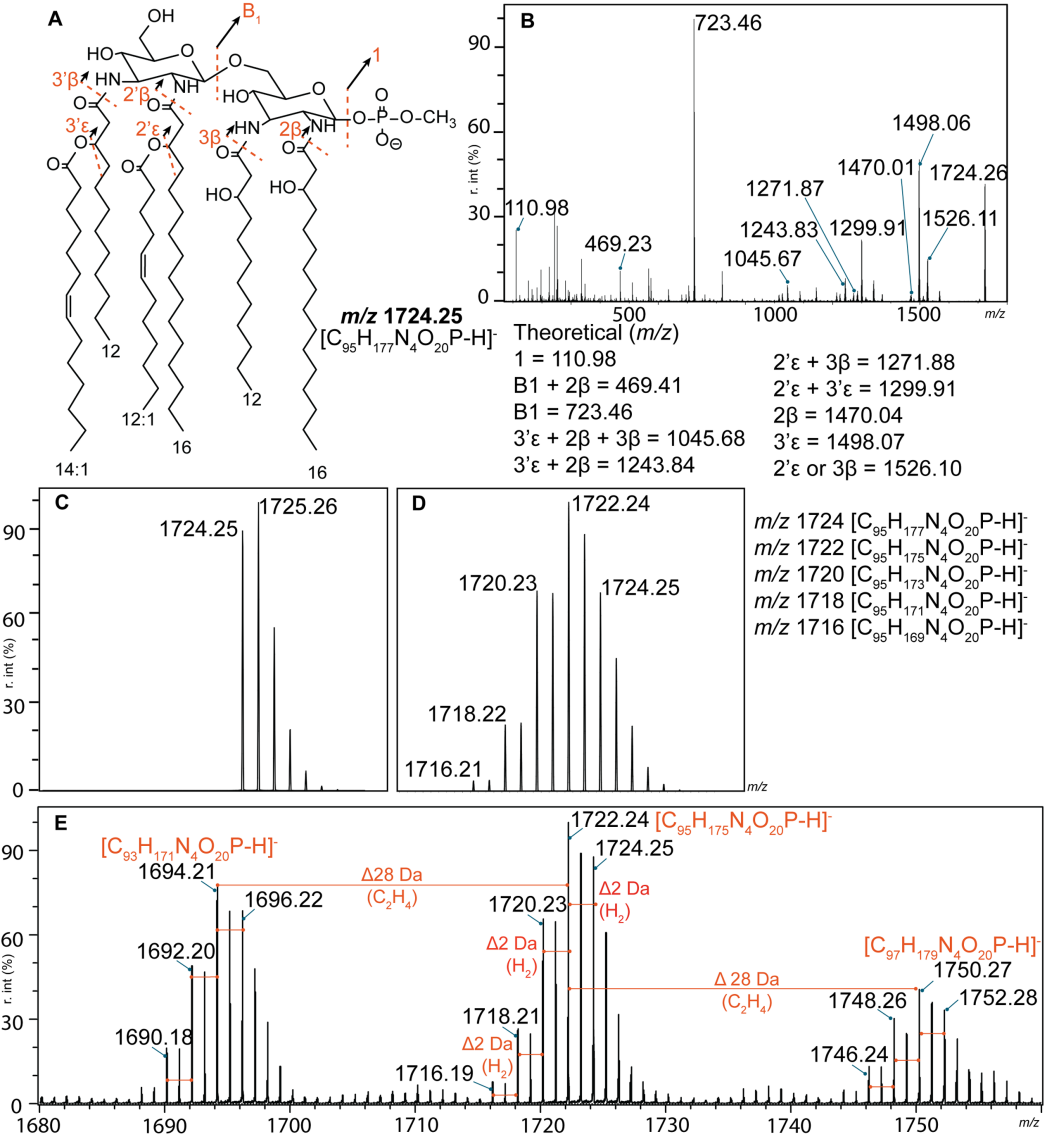
Structural determination of *L. interrogans* lipid A (L1) by tandem mass spectrometry. **(A)** Proposed structure of the *m/z* 1724.25 ion corresponds to the previously published structure for this species (Que-Gewirth et al., 2004; Eshghi et al., 2015). Fragmentation patterns are depicted as dashed red lines. **(B)** Product ion scan of the precursor ion *m/z* 1724.25. Calculated *m/z* of the product ions are listed at the bottom of the panel. **(C,D)** Theoretical isotopic distributions. **(C)** Isotopic distribution of a single lipid A ion (*m/z* 1724.25 corresponding to the structure in panel **A**). **(D)** Mixed isotopic distribution of five lipid A ions that differ from each other by a presence of a single double bond (2 Da). Please note that the abundances of each ion were not equal; ratio used for the simulation was 9:21:17:6:1 (*m/z* 1724:1722:1720:1718:1716). **(E)** Annotated mass spectrum of *L. interrogans* lipid A profile (L1). Three main clusters of lipid A ions separated by 28 Da were identified. Each individual cluster likely consisted of five lipid A ions that differ by a presence of a double bond (red lines). r. int. – relative intensity.

**Figure 3:**
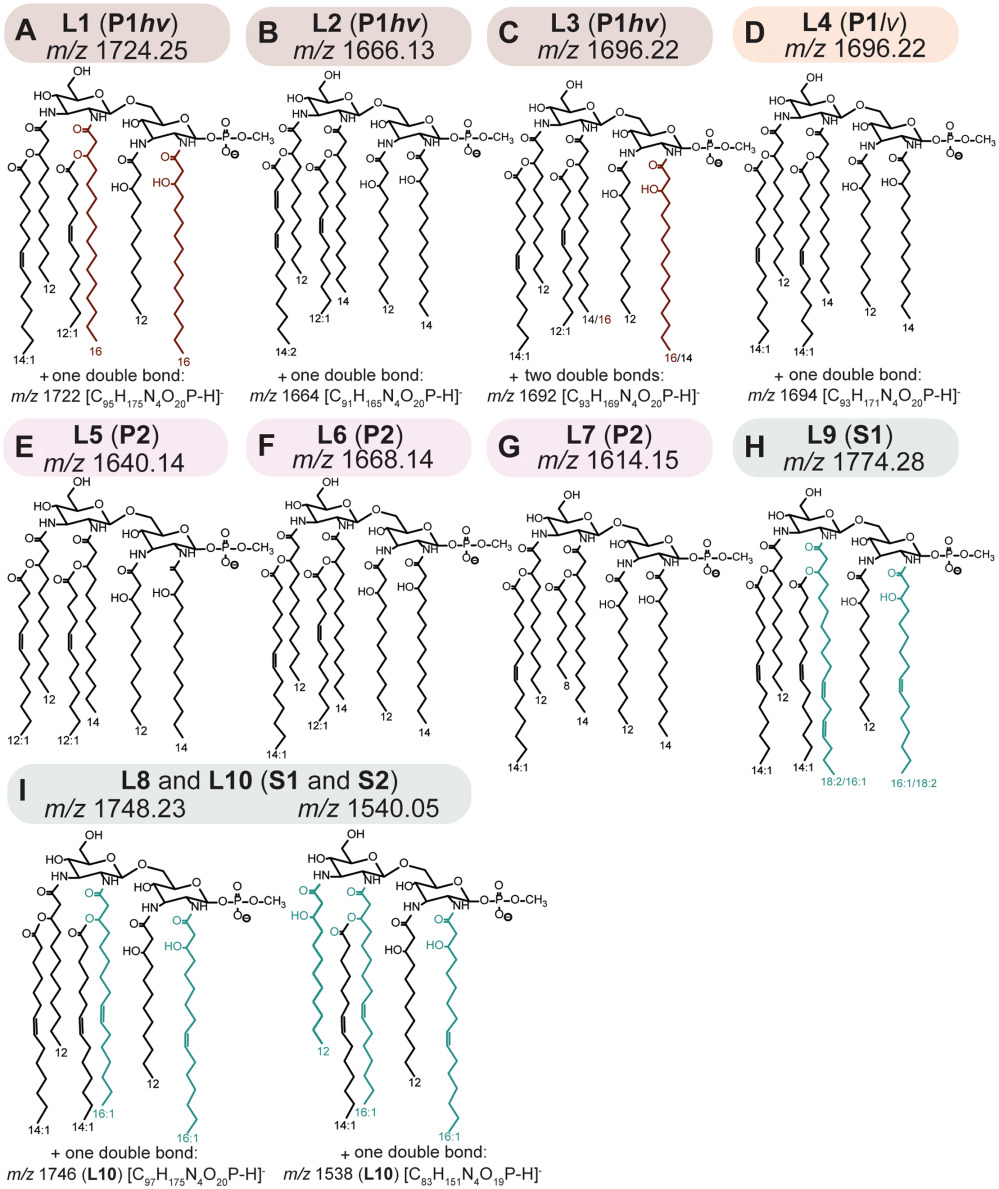
(A–C) L1-L3; P1hv group. (D) L4; P1lv group. **(E–G)** L5-L7; P2 subclade. **(H)** L9; S1 subclade. **(I)** L8 and L10; S1 and S2 subclades. Putative structures of lipid A ions representative of the L1–L10 profiles. Structures were proposed based on fragmentation patterns of the representative lipid A ions, as detected by FLAT*^n^* (Yang et al., 2022a; Supplementary Figures S4–S6). Sugar and anomeric configurations were assigned in homology with previous data (Que-Gewirth et al., 2004; Eshghi et al., 2015; Novak et al., 2022) and the placement of double bonds is putative. Additional approaches discussed in Section 3.2 are needed to fully validate the proposed structures (out of the scope of the current study). Please refer to the Table 2 for information on distribution of the individual phenotype across *Leptospira* species.

The structures of the representative lipid A ions of each profile (L1–L10) were proposed based on tandem mass spectrometry analysis (FLAT*^n^*) (Yang et al., 2022a). The lipid A structure of *L. interrogans* (L1) corresponded to the previously reported structure for this species (Que-Gewirth et al., 2004; Eshghi et al., 2015), validating our methodology (Figures 2A,B). Interpretation of lipid A profiles can be challenging. However, one main lipid A ion is usually surrounded by satellite molecules resulting from substantial modifications to this lipid A molecule (such as addition of a sugar moiety or a terminal phosphate group) (Leung et al., 2017; Liang et al., 2019). In contrast, all *Leptospira* lipid A profiles were complex with several clusters of lipid A ions separated by 26 or 28 Da (Figures 1, 2E). These mass differences corresponded to an addition of two carbons connected by a double bond or a single bond, respectively, and were previously described in *L. interrogans* and *L. kirschneri* (Novak et al., 2022) (Figure 2E). Each of these clusters was further predicted to consist of five individual lipid A ions separated by 2 Da (a double bond), revealing an unusual lipid A heterogeneity within a single bacterial species (Figure 2E). Briefly, if only a single lipid A ion was present, the isotopic distribution would look as depicted in Figure 2C. Instead, the measured isotopic distribution in each lipid A cluster (Figure 2E) closely corresponded to a mixed isotopic distribution consisting of five lipid A ions differentiated by a presence of a double bond (Figure 2D). Mass spectrometry-based strategies to locate positions of double bonds in unsaturated lipid molecules exist. They include chemical derivatization prior mass spectrometry analysis, and are yet to be tested on complex mixtures of lipid A molecules detected in *Leptospira* species (Figure 1E; Novak et al., 2022). Alternatively, proposed lipid A structures can be supported with other analytical techniques, such as nuclear magnetic resonance (NMR). However, dissolving lipid A in NMR-compatible solvents is challenging due to its amphipathic nature (Ribeiro et al., 1999; Zähringer et al., 2001; Silipo et al., 2002). The NMR approach is therefore more appropriate for characterization of the water-soluble components of LPS (core oligosaccharide and O-antigen). Both above-mentioned strategies require pure lipid A extracts from large volume of *Leptospira* culture, chemical derivatization reagents and rigorous method optimization for complex lipid A samples. Localization of double bonds was therefore not possible within the scope of this study. Like others (Eshghi et al., 2015; Novak et al., 2022) we therefore proposed structures of the representative lipid A for each of the lipid A profiles (L1–L10), and concluded that additional degrees of unsaturation were present (Figure 3). It is important to note that our approach allowed us to obtain valid structural information on *Leptospira* lipid A from an equivalent of 10^7^ cells (approximately 100 μL of exponential culture). Experiments were therefore performed in a controlled manner, using biological triplicates on two independent experimental days. The low amount of starting material does not affect the results. Lipid A structures of *Pseudomonas aeruginosa, Acinetobacter baumannii,* and *Klebsiella pneumoniae* proposed by FLAT*^n^* corresponded to those determined by other methodologies (Yang et al., 2022a). Here we also validated FLAT*^n^* on lipid A of *L. interrogans* serovar Manilae strain L495 (Figure 1A; Eshghi et al., 2015).

### 3.3. The interspecies variability of *Leptospira* lipid A: 2 and 2’primary acyl chains

Tandem MS analysis revealed that some previously described *Leptospira* lipid A features (Que-Gewirth et al., 2004; Eshghi et al., 2015) were conserved in all 31 examined species. Specifically, representative lipid A molecules were hexa-acylated and monophosphorylated, with all primary fatty acyl residues linked *via* amide bonds and a methylated terminal phosphate group present at 1 position of the di-aminoglucose backbone (Figure 3). A newly discovered conserved feature was the presence of C12:0 (OH) residues at the 3 and 3′ primary positions (Figure 3).

The structural variability of lipid A between the *Leptospira* species was determined by the length and saturation of 2 and 2′ primary acyl and 2′ and 3′ secondary acyl chains (Figure 3). The identity of 2 primary acyl chains could be deducted from the main product B_1_ ion that results from fragmentation of the bond connecting the glucosamine backbone (Figures 2A,B). The three main B_1_ ions identified in this study were *m/z* 695, *m/z* 721 and *m/z* 723 indicating acylation of the phosphorylated sugar unit (Glc*N* I) at position 2 with C14:0 (OH), C16:1 (OH) and C16:0 (OH), respectively (Supplementary Figures S4–S6). The 2 and 2′ primary acyl chains are usually identical, which stems from the mechanism of the lipid A biosynthesis (Raetz et al., 2009; Simpson and Trent, 2019). Lipid A is synthesized in a series of conserved reactions mediated by the family of Lpx enzymes; homologs of most Lpx enzymes were identified across *Leptospira* species (Hinckley et al., 2005; Eshghi et al., 2015; Nieves et al., 2023). In the early steps, LpxA, LpxC, and LpxD produce a molecule of uridine phosphate (UDP)-2,3-diacylglucosamine from UDP-N-acetylglucosamine and fatty acids bound to acyl carrier proteins. While LpxA is responsible for the addition of a fatty acyl to the 3 primary position, LpxD adds a fatty acyl to the 2 primary position of the glucosamine backbone. Both LpxA and LpxD have affinity toward specific fatty acyl chains, and this affinity differs across bacterial species (Simpson and Trent, 2019). Subsequently, a molecule of “lipid X” (2,3-diacylglucosamine-1-phoshate) is produced from some UDP-2,3-diacylglucosamine precursors *via* activity of LpxH or its homologs LpxI or LpxG. One UDP-2,3-diacylglucosamine and one “lipid X” molecule are then condensed together *via* the activity of LpxB, resulting in identical acyl chains in the 2 and 2′ and in the 3 and 3′ primary positions. As follows, each of the individual L*eptospira* subclades had a predominant primary acyl chain at the 2 and 2′ primary positions: C16:0 (OH) acyls were detected exclusively in P1*hv* species (Figures 3A,C), C14:0 (OH) in all P1*lv* and P2 species (Figures 3D–G) and C16:1 (OH) in all S1 and S2 species (Figures 3H,I). This was consistent with a previous study where C16 (OH) were detected exclusively in the pathogenic *L. interrogans* (Patra et al., 2015). Lipid A of *L. mayottensis* incorporated two C14:0 (OH) as the 2 and 2′ primary residues and its lipid A therefore resembled those of the P1*lv* and P2 species (Figure 3B, L2 profile).

Interestingly, fragmentation of the L3 and L9 representative lipid A ions resulted in two B_1_ ions instead of one (Supplementary Figures S4D, S6F). In L3 (*L. santarosai*), the two B_1_ ions *m/z* 695 and *m/z* 723 were detected, suggesting that the 2 and 2′ primary acyl chains were interchangeable, creating two possible isomers. A combination of C14:0 (OH)/C16:0 (OH) at the 2/2′ positions resulted in the *m/z* 695 B_1_ product ion, while the opposite configuration, C16:0 (OH)/C14:0 (OH) at the 2/2′ positions, resulted in the *m/z* 723 B_1_ ion (Figure 3C). The lipid A profile of this strain was also the most complex one with two extra double bonds in the base lipid A ion that could not be localized using the MS data alone (Figure 3C). In L9 (*L. kanakyensis* and *L. noumeaensis*), *m/z* 721 and *m/z* 747 were detected, likely resulting from combinations of C16:1 (OH)/C18:2 (OH) and C18:2 (OH)/C16:1 (OH) at the 2/2′ primary positions, respectively (Figure 3H). These unusual lipid A structures could be a result of simultaneous activity of two LpxD enzymes, as two copies of *lpxD* genes have been annotated in all *Leptospira* genomes except for those belonging to the P2 subclade (Supplementary Figure S7). To date, the function of two separate LpxD enzymes was studied only in two bacterial species (Simpson and Trent, 2019). In *Francisella*, the expression of LpxD1 and LpxD2 is temperature dependent. LpxD1 adds two C18:0 (OH) and LpxD2 adds two C16:0 (OH) to the 3 and 3′ primary positions of the lipid A when grown at 37°C and 25°C, respectively, aiding adaptation to temperature shifts (Gunn and Ernst, 2007; Scott et al., 2016). In *L. interrogans*, LpxD1 contributes to pathogenicity, adaptation to temperature changes and presence of toxic compounds (Eshghi et al., 2015). However, the conditions warranting expression of LpxD1/LpxD2 in *L. interrogans* remain elusive (Eshghi et al., 2015; Simpson and Trent, 2019). The representative structures of L3 and L9 phenotypes might provide a first hint to function of LpxD1 and LpxD2 in other *Leptospira* species. It occurs that *Leptospira* species with the L3 and the L9 phenotypes, co-expressed LpxD1 and LpxD2 enzymes might compete to add acyl residues to the 2 primary position of the early UDP-2,3-diacyl glucosamine product in lipid A biosynthesis. A similar phenomenon was described for the late acetyltransferases LpxL1 and LpxL2 in *Klebsiella pneumoniae* that compete to add either C12:0 or C14:0 at the 2′ secondary position (Li et al., 2016; Mills et al., 2017; Simpson and Trent, 2019). Annotated tandem mass spectra for all representative lipid A ions can be found in the supplementary material (Supplementary Figures S4–S6).

### 3.4. The interspecies variability of *Leptospira* lipid A: 2′ And 3′ secondary acyl chains

All *Leptospira* species incorporated short fatty acyl chains at the 2′ and 3′ secondary positions of their lipid A. In the representative lipid A molecules, these residues consisted of a combination of C12:1/C14:1 (L1, L3 and L6; Figures 3A,C,F), two C12:1 (L5; Figure 3E) or two C14:1 (L4, L8-10; Figures 3D,H,I). Representative lipid A molecules of *L. mayottensis* (L2; Figure 3B) and *L. perolatii* (L7; Figure 3G) contained a combination of C14:2/C12:1 and C14:1/C8:0, respectively (Figure 3). Secondary acyl residues are added to the lipid A by late acyltransferases (homologs of LpxL and LpxM from *Escherichia coli*) (Raetz et al., 2009). Each of these enzymes often adds an acyl chain of a specific length and degree of saturation (Simpson and Trent, 2019). To date, only one bi-functional acyltransferase capable of adding two different acyl chains to the 2′ and 3′ secondary positions was reported in *Acinetobacter baumannii* (Boll et al., 2015). Given the great variability of secondary acyl chains across *Leptospira*, its LpxL homolog is likely a multifunctional acyltransferase.

### 3.5. Penta-acylated lipid A molecules were detected in S1 and S2 *Leptospira* species

A novel structural feature of *Leptospira* lipid A was found in S1 and S2 subclades. In these species, additional clusters with lower *m/z* were identified (Figure 1; Supplementary Figure S3). Upon tandem mass spectrometry analysis, it was determined that these were penta-acylated lipid A molecules (Figure 3; Supplementary Figure S6). The mechanisms of synthesis of these penta-acylated lipid A species are unclear. In other bacteria, fatty acyl chains can be removed *via* activity of LpxR (Simpson and Trent, 2019). Although homologs of LpxR were identified in *Leptospira*, the LpxR usually removes two acyl chains, not one. PagL and PagP enzymes can remove a single acyl chain from the lipid A molecule (Ernst et al., 2006; Thaipisuttikul et al., 2014), however, homologs of these enzymes were not found in saprophytic *Leptospira* (Picardeau et al., 2008). Finally, in bacteria harboring two LpxL enzymes, such as *Neisseria meningitidis*, loss of one copy leads to synthesis of penta-acylated lipid A species (Fransen et al., 2009). This cannot be the case in *Leptospira* where only one LpxL homolog was annotated (Picardeau et al., 2008). Nonetheless, penta-acylated lipid A molecules are known to elicit reduced immune responses in the host (Fransen et al., 2010; Scott et al., 2017), and their presence in saprophytic species is intriguing.

### 3.6. Which structural features of *Leptospira* lipid A might contribute to pathogenicity?

Lipid A of pathogenic *Leptospira* has been previously shown to evade recognition by the human TLR4/MD2 (Werts et al., 2001). It has been speculated that this might be due to monophosphorylation of *Leptospira* lipid A that is associated with a reduced endotoxic activity in other bacteria (Baldridge and Crane, 1999; Wang et al., 2007). However, this is complicated by the unusual presence of a methyl group on the single terminal phosphate (Que-Gewirth et al., 2004). Here, we revealed other structural features that might contribute to this phenomenon.

Degree of TLR4/MD2 activation is also dependent on the length of fatty acyl residues. While C12 or C14 are optimal for TLR4/MD2 binding, C16 is not favorable (Rietschel et al., 1994; Park and Lee, 2013; Facchini et al., 2018). In deep-sea *Moritella* species, the lipid A either activates TLR4/MD2 or is “immune-silent,” not eliciting responses *via* TLR4/MD2 or other related host receptors (Gauthier et al., 2021). While the basic structural features between *Moritella* lipid As are conserved (hexa-acylated bis-phosphorylated molecules), the immune-silent *Moritella* lipid A has higher C16 content (Gauthier et al., 2021). In this study, C16 (OH) residues were found exclusively in pathogenic P1*hv* species (Figures 3A,C), which was consistent with previous findings (Patra et al., 2015). We therefore hypothesize that lipid A of P1*lv* and P2 species might be better binding partners of the innate immune receptors, contributing to faster clearance of these species and their lower pathogenic potential in humans. Future studies including assessing endotoxin activity of P1*lv* and P2 lipid A extracts using reporter assays are warranted to explore this hypothesis.

Finally, while the discussion to this point has centered around lipid A, other lipid molecules are known to confer immune evasion. Cardiolipins have been shown to suppress stimulatory activity of LPS (Khan et al., 2018). Cardiolipin species have been identified in pathogenic as well as non-pathogenic *Leptospira* species (Supplementary Figure S8). Since our lipid preparations for FLAT and FLAT*^n^* consisted of whole cells, it is not possible to determine if the cardiolipins were located to the inner or the outer membrane and if they can attenuate LPS-mediated immune activation. However, their presence in the *Leptospira* membrane is intriguing and warrants further investigation.

### 3.7. Lipid A-based molecular typing as a complementary strategy for *Leptospira* identification and classification

Novel *Leptospira* species are isolated from various hosts or the environment on regular basis (Thibeaux et al., 2018; Masuzawa et al., 2019b; Korba et al., 2021). Extensive phenotype profiling including serotyping, assessing growth at 37°C, growth in presence of purine analog 8-azaguanine, and ultimately animal infection studies are needed to distinguish pathogens from saprophytes during characterization of novel species (Vincent et al., 2019). Here, we propose the use of L1-L10 lipid A profiles combined with FLAT for rapid classification of *Leptospira* isolates into the individual subclades (L1-L3 for P1*hv*, L4 for P1*lv*, L5-L7 for P2, L8-9 for S1 and L10 for S2 subclades). Lipid A-based MALDI-TOF assays allow for rapid (within an hour) identification of bacteria directly from a specimen using minimal input and hands-on-time (Leung et al., 2017; Liang et al., 2019; Sorensen et al., 2020). Lipid A-based assays allow for simultaneous identification and screening for antibiotic resistance markers and can be used directly from urine (Smith et al., 2021, 2022; Yang et al., 2022b). Thanks to minimal background in the *m/z* area where lipid A is detected, individual species can also be identified from multi-bacterial samples (Fondrie et al., 2018; Ryu et al., 2020). Protein-based profiling *via* MALDI-TOF is routinely used to characterize *Leptospira* species (Thibeaux et al., 2018; Sonthayanon et al., 2019; Girault et al., 2020; Korba et al., 2021), and the addition of lipid A phenotyping would provide valuable information while utilizing the existing infrastructure.

## 4. Conclusion

This is the first study focused on structural analysis of lipid A across the whole *Leptospira* genus. Ten distinct lipid A profiles were revealed that can be used for rapid molecular typing of novel clinical and environmental *Leptospira* isolates, aiding the leptospirosis surveillance. In addition, revealed structural differences between lipid A of individual species can lead to novel hypotheses on *Leptospira* pathogenicity.

## Data availability statement

The original contributions presented in the study are included in the article/Supplementary material, further inquiries can be directed to the corresponding authors.

## Author contributions

HP conceived and designed the experiments. HP, AM, SC, AG-G, and MS performed the experiments. HP, AM, and DG analyzed the data. AG, RE, CC, MP, and DG contributed reagents, materials, and analysis tools. HP and DG prepared the original draft. HP, AM, SC, AG-G, AG, MS, RE, CC, MP, and DG reviewed and edited the manuscript. All authors contributed to the article and approved the submitted version.

## Funding

Funding for this study was provided to DRG from Natural Sciences and Engineering Research Council of Canada (NSERC) Discovery Grant (RGPIN-2022-04433). DG and RE received funding from National Institutes of Health (NIH) and National Institute of Allergy and Infectious Diseases (NIAID) #R01AI147314. DG and CC received support from the Canada Institutes of Health Research (CIHR), Catalyst Grant Sexually Transmitted and Blood Borne Infections Research in Canada: Beyond HIV/AIDS and Hepatitis (SC1–178732). DG and the work performed at the University of Victoria-Genome BC Proteomics Centre was also supported by funding to the Metabolomics Innovation Centre (TMIC) from Genome Canada and Genome British Columbia, through the Genomics Technology Platform (GTP) program for operations and technology development (265MET), as well operations support from the Canadian Foundation for Innovation Major Sciences Initiative (CFI-MSI) program (35456). Infrastructure and operations funding to support his project was provided to DRG from PacifiCan (Project No: 22591). MP was supported by Institut Pasteur.

## Conflict of interest

RE and DG are co-founders and vice presidents of Patagain, a company that develops mass spectrometry-based microbiology tests to identify disease pathogens and determine antimicrobial resistance.

The remaining authors declare that the research was conducted in the absence of any commercial or financial relationships that could be construed as a potential conflict of interest.

## Publisher’s note

All claims expressed in this article are solely those of the authors and do not necessarily represent those of their affiliated organizations, or those of the publisher, the editors and the reviewers. Any product that may be evaluated in this article, or claim that may be made by its manufacturer, is not guaranteed or endorsed by the publisher.
