# Supplementary material for: Lipid A structural diversity among members of the genus Leptospira
Source: Front Microbiol. 2023 May 25;14:1181034. doi: 10.3389/fmicb.2023.1181034 (PMC10248169; doi:10.3389/fmicb.2023.1181034)
Supplement: Supplementary file 1 [file Data_Sheet_1.PDF]

SUPPLEMENTARY FIGURES

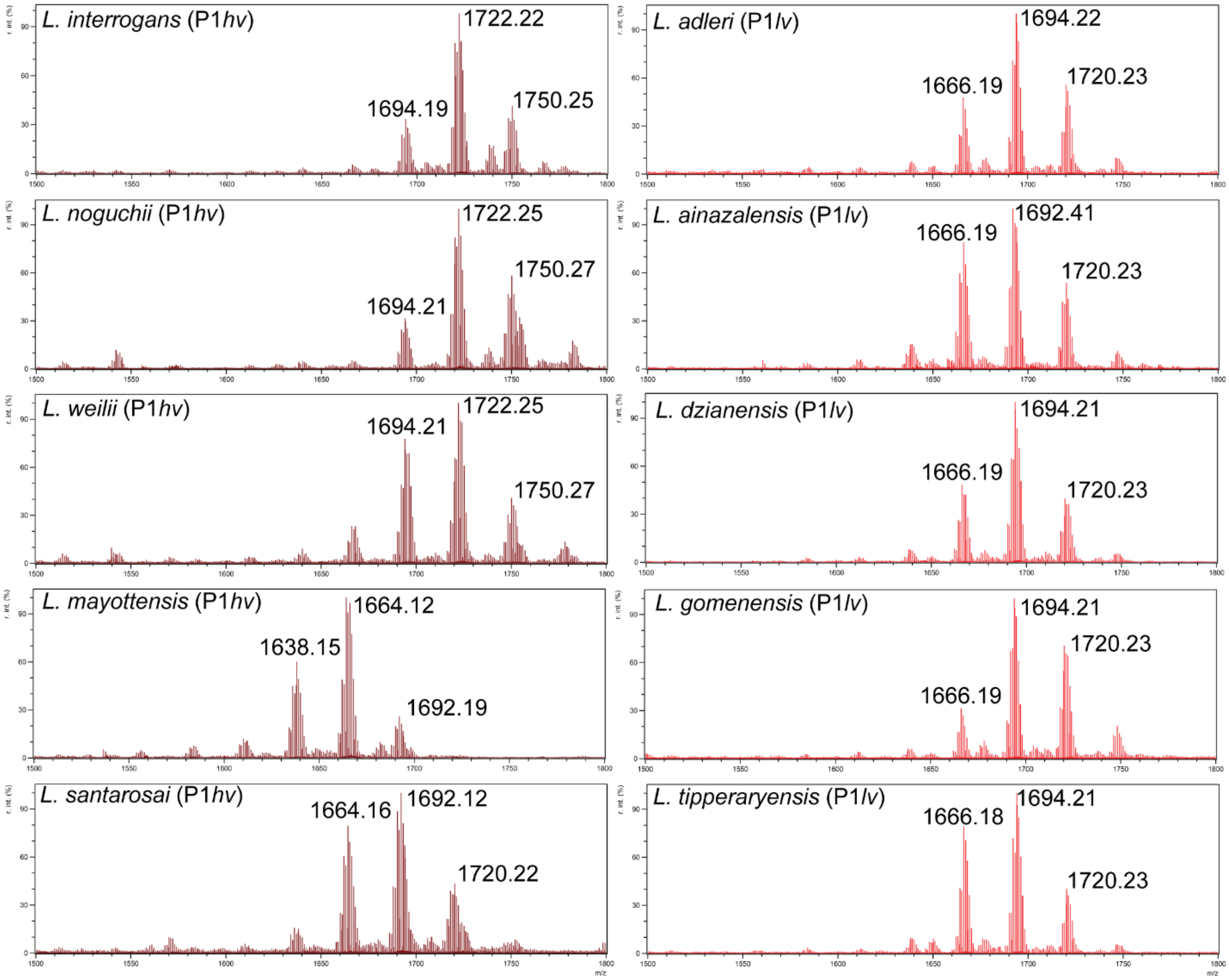

Fig. S1. Lipid A profiles of the individual P1*hv* and P1*lv* species. r. int. – relative intensity.

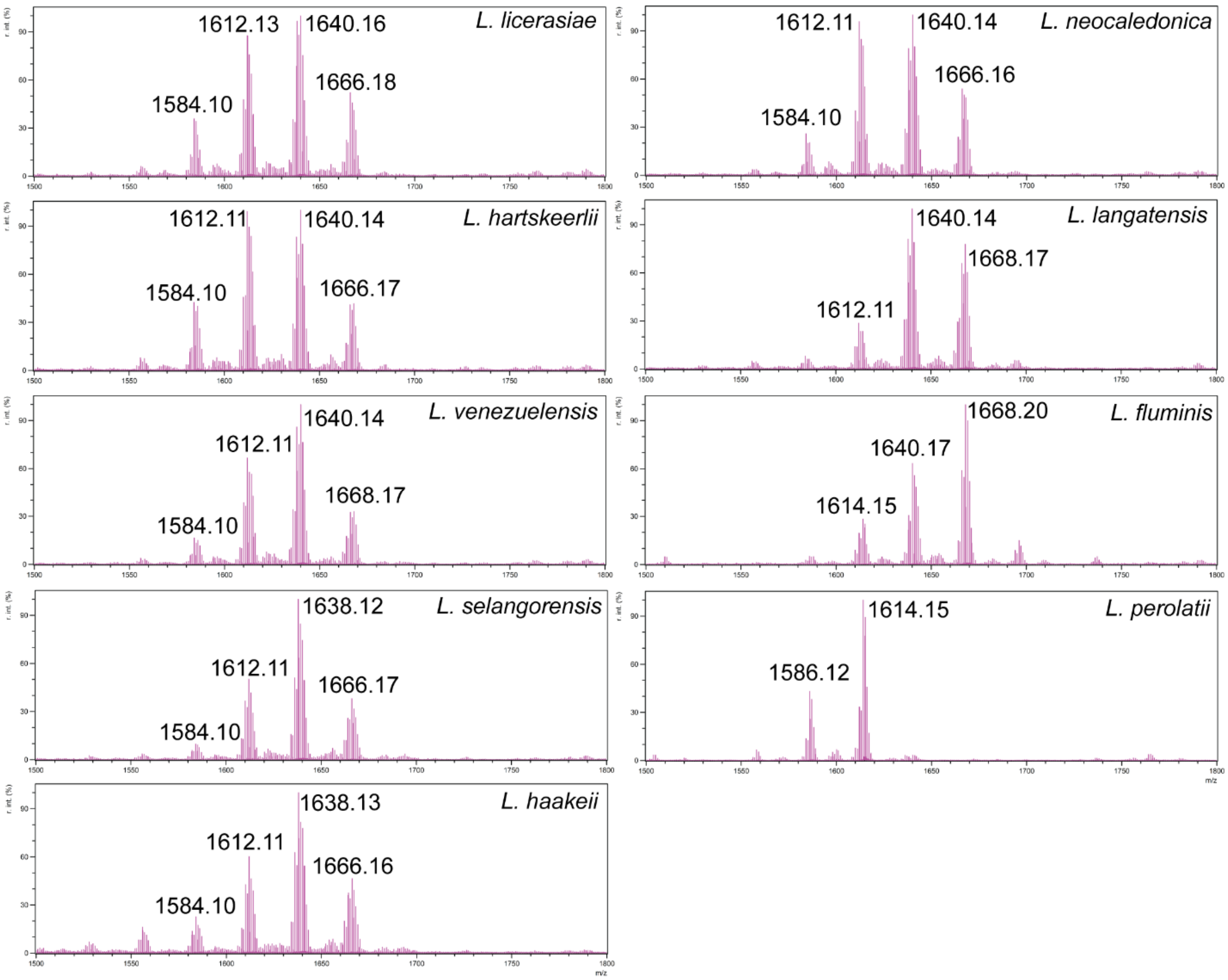

Fig. S2. Lipid A profiles of the individual P2 species. r. int. – relative intensity.

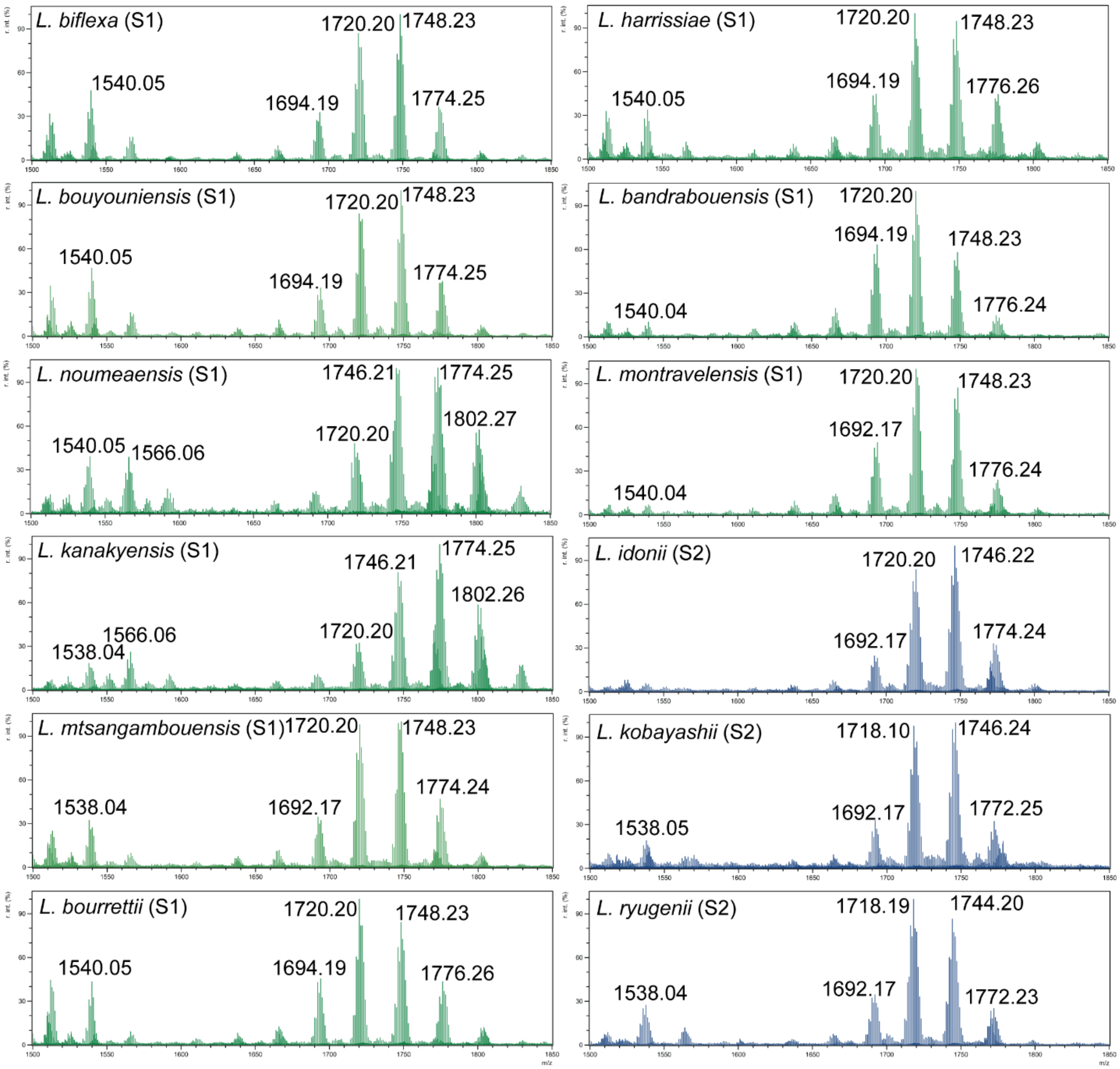

Fig. S3. Lipid A profiles of the individual S1 and S2 species. r. int. – relative intensity.

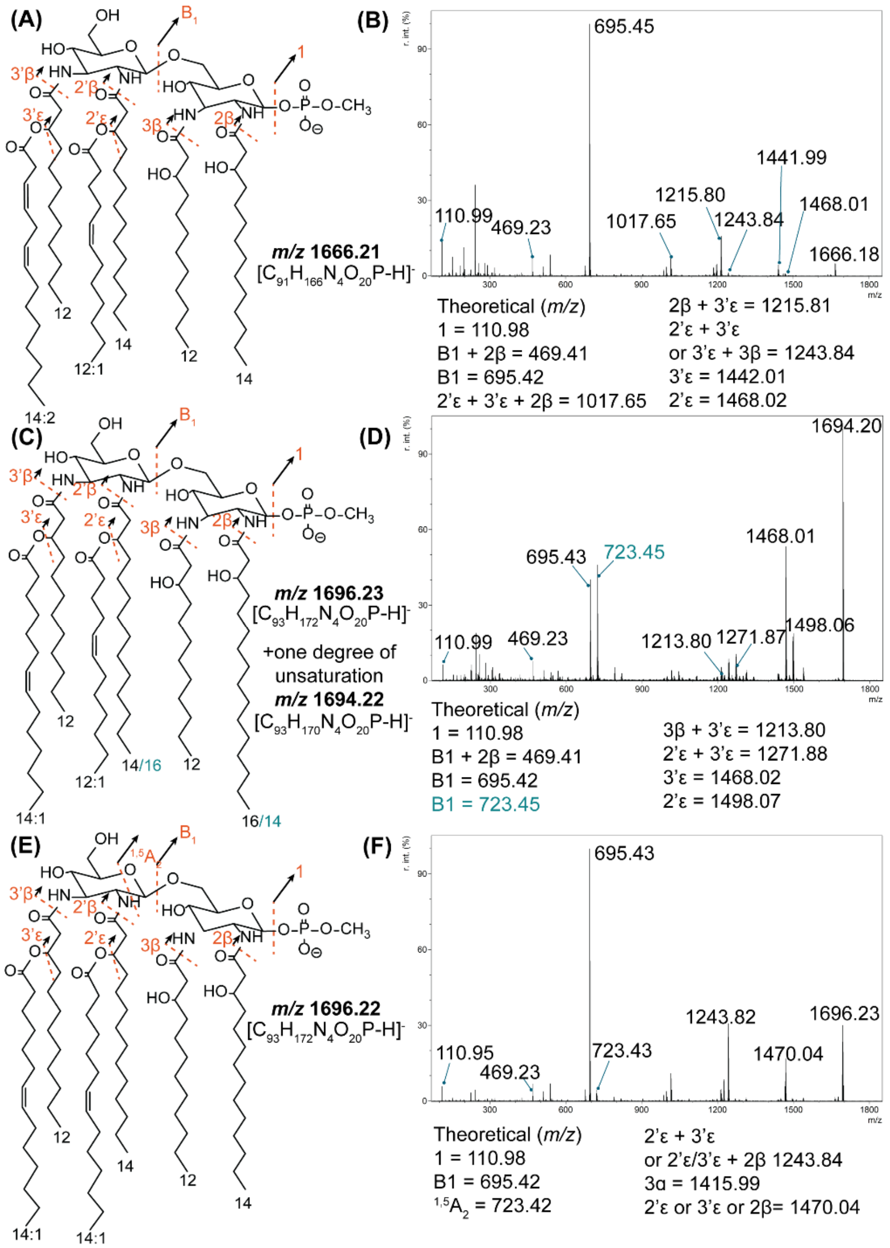

Figure S4. Fragmentation patterns and the corresponding tandem mass spectra of the representative lipid A ions in P1 *Leptospira* species. A-B. *m/z* 1666, L2 profile. **C-D.** *m/z* 1696, L3 profile. Please note that the (fragmented) parent ion was *m/z* 1694 (**D**) while the predicted structure is representative of *m/z* 1696 (**C**). Based on the fragmentation patter alone, it was not possible to determine the placement of the extra double bond. The two product B_1 ions were likely a result of mismatched 2 and 2’ primary acyl chains. A combination of C16:0 (OH)/C14:0 at the 2/2’ resulted in the *m/z* 695 product ion, while a combination of C14:0 (OH)/C16:0 (OH) at the 2/2’ resulted in the *m/z* 723 product ion (corresponding acyl chains position and B_1 annotation shown in blue). **E-F.** *m/z* 1696, L4 profile r. int. – relative intensity.

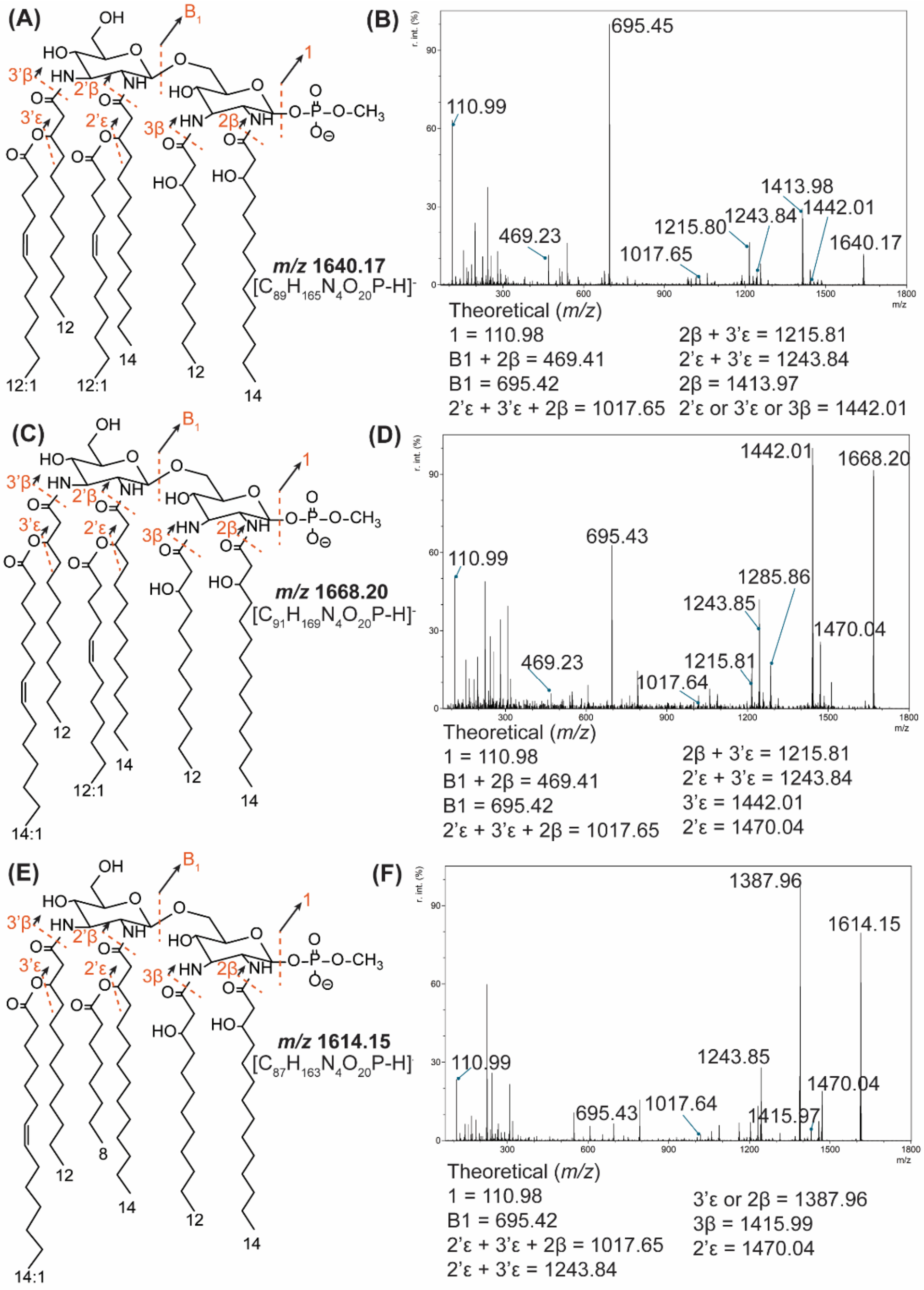

Figure S5. Fragmentation patterns and the corresponding tandem mass spectra of the representative lipid A ions in P2 *Leptospira* species. A-B. *m/z* 1640, L5 profile. **C-D.** *m/z* 1668, L6 profile. **E-F.** *m/z* 1614, L7 profile r. int. – relative intensity.

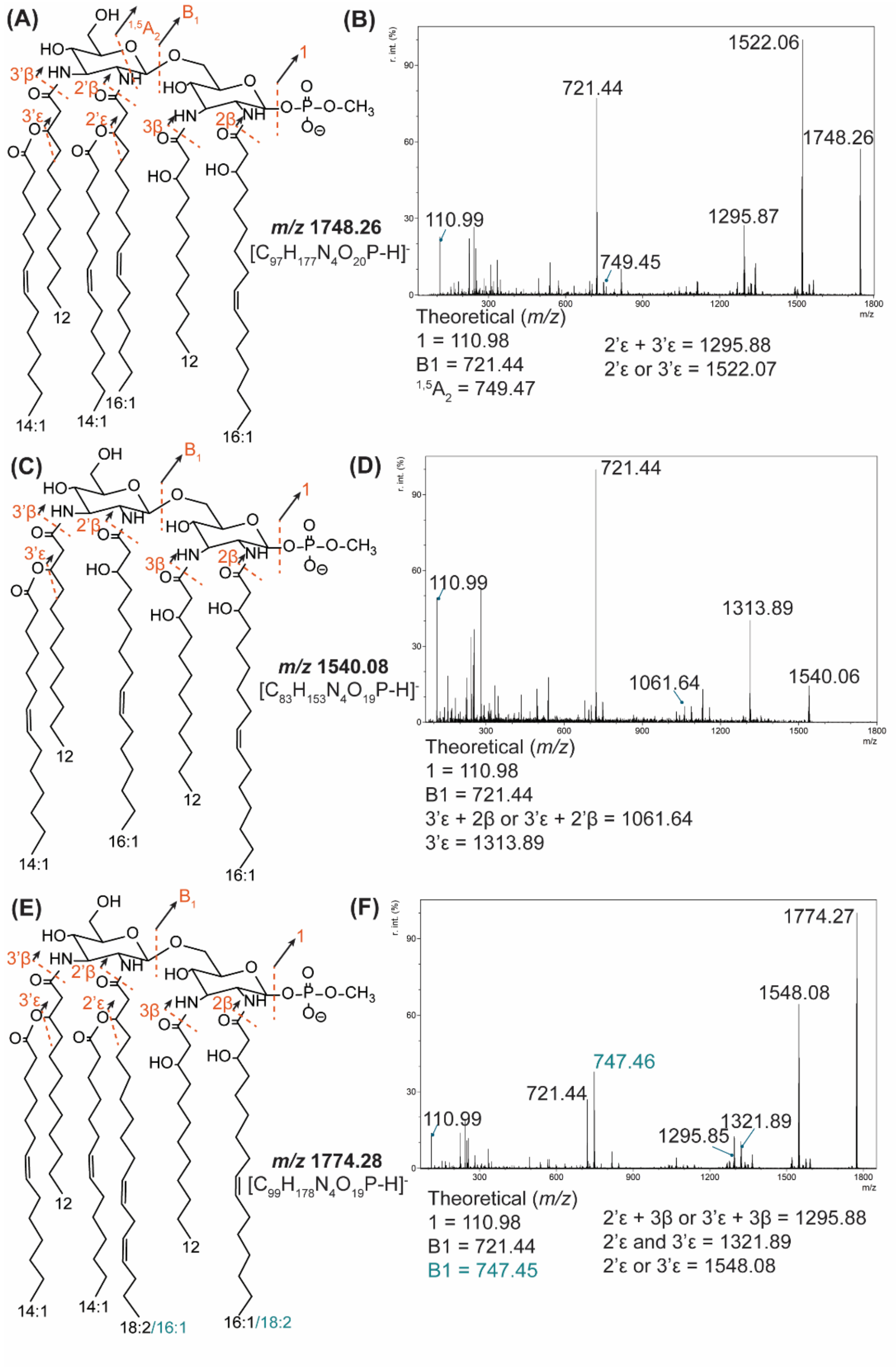

Figure S6. Fragmentation patterns and the corresponding tandem mass spectra of the representative lipid A ions in P1 *Leptospira* species. A-B. *m/z* 1748, L8 and L10 profile. **C-D.** *m/z* 1540, L8 and L10 profile. **E-F.** *m/z* 1774, L9 profile. The two product B_1 ions were likely a result of mismatched 2 and 2’ primary acyl chains. A combination of C16:1 (OH)/C18:2 at the 2/2’ resulted in the *m/z* 721 product ion, while a combination of C18:2 (OH)/C16:1 (OH) at the 2/2’ resulted in the *m/z* 747 product ion (corresponding acyl chains position and B_1 annotation shown in blue). r. int. – relative intensity.

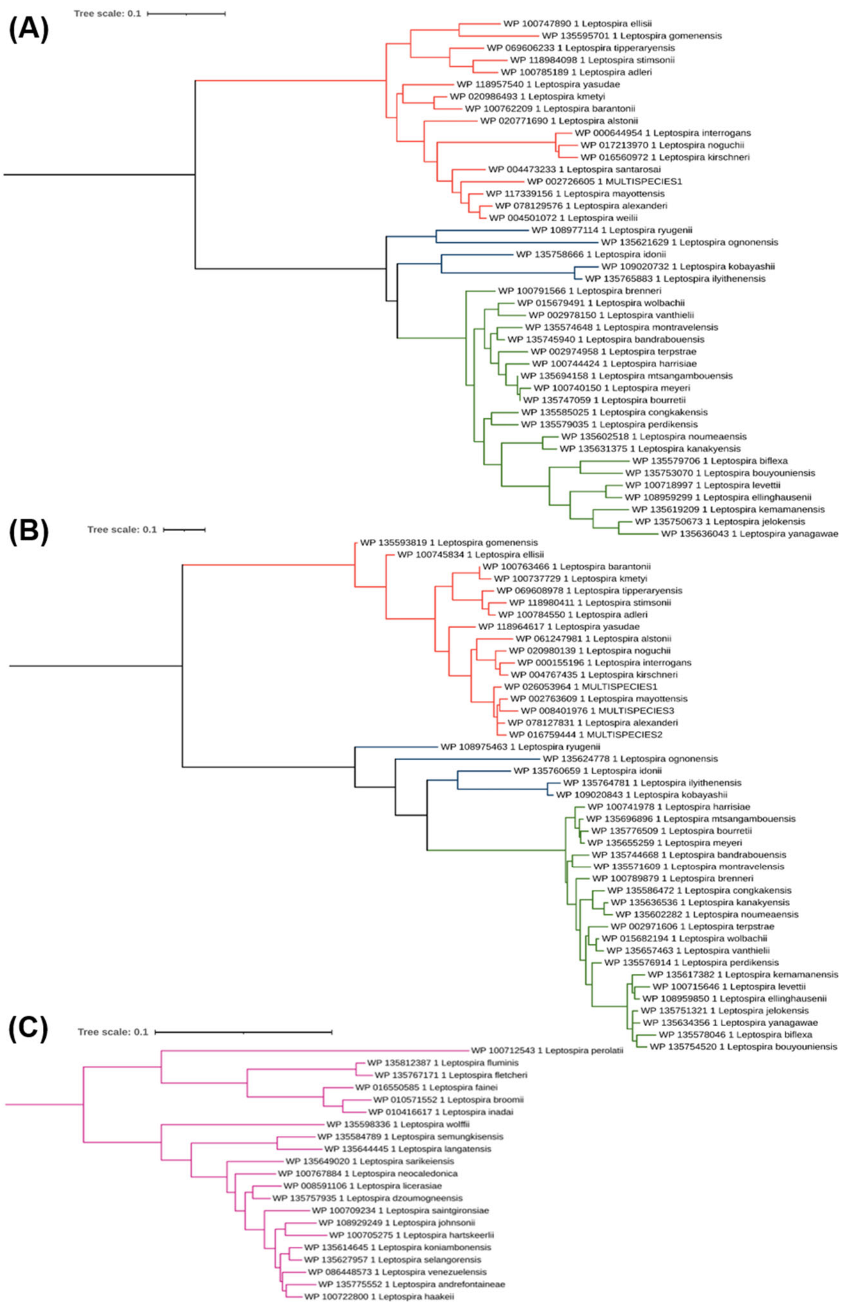

Figure S7. Phylogenetic trees based on *Leptospira* LpxD protein sequences. Reference protein sequences were downloaded from the GenBank database (WP numbers included in the figure). Trees were created in NGPhylogeny.fr [35] using MAFFT alignment with BMGE curation, PhyML tree interface and Newick tree rendering. **A.** LpxD1 in P1, S1 and S2 subclades. MULTISPECIES1: *L. borgpetersenii*. **B.** LpxD2 in P1, S1 and S2 subclades. MULTISPECIES: *L. borgpetersenii* (1), *L. weilii* (2) and *L. santarosai* (3). **C.** LpxD (P2 subclade only). P1subclade – red, P2 subclade – purple, S1 subclade – green, S2 subclade – blue.

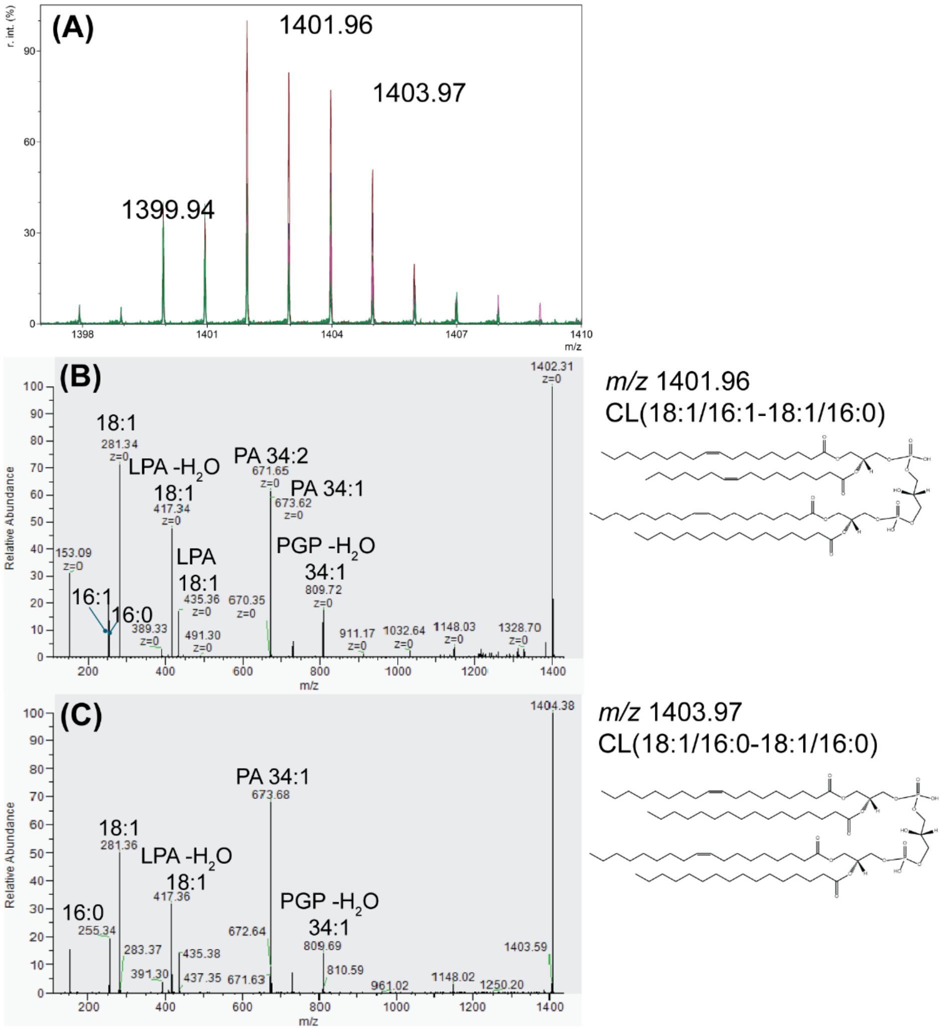

Figure S8. Cardiolipin species detected in *Leptospira*. (A) MS1 profiles of cardiolipins (CL) in *Leptospira*. MS1 scan is shown overlay; dark red (P1*hv*), light red (P1*lv*), purple (P2), green (S1) and blue (S2). Data were obtained post FLAT in the negative ion mode on the timsTOF *flex* MALDI-2 (Bruker, Bremen, Germany). **(B-C) Tandem MS data.** Cell pellets of *L. interrogans* were mixed with 1:3 v/v chloroform/methanol, vortexed, sonicated, and centrifuged. Supernatants were transferred into autosampler vials, dried and resolubilized in 1:1 v/v chloroform/methanol. HPLC-MSMS analysis was performed on an Ultimate 3000 UPLC system coupled to a Thermo LTQ-Orbitrap Velos Pro mass spectrometer (Thermo Fisher Scientific, Waltman, MA, USA). Lipids were injected on a Phenomenex Jupiter C4 column (40 °C). The mobile phases were (A) 1 mM ammonium acetate and (B) 98% propanol/1 mM ammonium acetate, with the following gradient (min:%B): 0-10:30; 12:100; 12.1:100; 16:30. The HPLC-MSMS analyses were carried out with full-mass detection in the Fourier transform MS mode (FTMS), with negative-ion detection. Fragmentation product ion masses were measured in the orbitrap using Higher energy collision-induced dissociation (65%) activation energy. Data were analyzed in Freestyle (Thermo Fisher). **(B)** CL 68:3 (m/z 1401.96): fragmentation pattern (left) and a predicted structure (right). **(C)** CL 68:2 (m/z 1403.97): fragmentation pattern (left) and a predicted structure (right). Fragmentation patterns by MALDI (FLAT^{n}) in representative species of all clades yielded identical results. LPA – lysophosphatidic acid. PA – phosphatidic acid, PGP – phosphatidylglycerolphosphate.
